# TREATMENT WITH ANTIPSYCHOTICS AND RISK OF DEATH IN 58,000 PATIENTS FROM SWEDISH DEMENTIA REGISTRY

**DOI:** 10.1093/geroni/igz038.1573

**Published:** 2019-11-08

**Authors:** Dorota Religa, Emilia Schwertner, sara Garcia-ptacek, Bjorn Johansson, Katarina Nägga, Bengt Winblad, Maria Eriksdotter, Juraj Secnik

**Affiliations:** 1 Karolinska Institutet, Stockholm, Sweden; 2 Clinical Memory Research Unit, Department of Clinical Sciences Malmö, Lund University, Malmö, Malmö, Sweden

## Abstract

Background: Behavioral and Psychological symptoms of Dementia (BPSD) are a heterogeneous group of clinical phenomena that is subjectively experienced by the patient and/or observable by an examiner (e.g., caregiver, physician) consisting in disturbed emotions, perception, thought, motor activity, and altered personality. There are recommendations to limit antipsychotic use in patients with dementia, and the big educational effort is made to follow them. In Sweden antipsychotics use changed from 10.1% in 2007-2008 to 5.2% in 2014-2015. Aims: The aim of the study is to analyze the actors associated with treatment and mortality of dementia patients, particularly those suffering from BPSD. Particularly we focus on assessing all-cause mortality patients with dementia treated with antipsychotic drugs (APDs). Methods: We have analyzed 58,412 patients newly diagnosed with dementia. We have found that 2526 of the patients were prescribed APDs (602 typical APDs and 1833 atypical APDs)- Results In the adjusted models, use of APDs at the time of dementia diagnosis was associated with increased mortality risk in the total cohort (hazard ratio = 1.4; 95% confidence interval 1.3-1.5). We have also stratified the results. Conclusions: The risk of death in patients with dementia was increased in group that used atypical and typical APDs. Our study gives more evidence to advice caution in APD prescription for patients with dementia.

